# Relationship between psychological stress with functional constipation in children: a systematic review

**DOI:** 10.11604/pamj.2023.46.8.41130

**Published:** 2023-09-07

**Authors:** Ferry Suganda Gozali, Beatrix Febiana, I Gusti Ngurah Sanjaya Putra, I Putu Gede Karyana, Badriul Hegar

**Affiliations:** 1 Department of Child Health, Faculty of Medicine Widya Mandala Catholic University, Surabaya, Indonesia,; 2 Department of Child Health, Faculty of Medicine Udayana University, I Goesti Ngoerah Gde Ngoerah Hospital, Denpasar, Indonesia,; 3 Department of Child Health, Faculty of Medicine Universitas Indonesia, Cipto Mangukusumo Hospital, Jakarta, Indonesia

**Keywords:** Children, functional constipation, pediatric, psychological stress

## Abstract

**Introduction:**

constipation affects up to 29.6% of children worldwide, making it one of the most common gastrointestinal illnesses in the pediatric population. As a functional disorder, the development of constipation is mostly influenced by a child´s psychosocial condition, even considered as one of important contributing factors. This systematic review aims to evaluate the relationship between psychological stress with constipation in the pediatric population.

**Methods:**

three online databases were searched as study sources, including PubMed, the Cochrane Library, and Google Scholar. Study selection was carried out using the PRISMA diagram. Studies that met the eligibility criteria were then included in the data extraction and synthesis. The study quality assessment was done using the Joanna Briggs Institute's (JBI) critical appraisal checklist.

**Results:**

eleven studies are included in this systematic review, consisting of four cross sectional studies, four case control studies and three cohort studies. The included studies have good quality based on the assessment. Majority of the studies showed a significant relationship between psychological stress and constipation in children. Psychological stress in children can be classified into family-related stressors, school-related stressors, exposure to stressful life events, stress related to psychological disorders, and other factors.

**Conclusion:**

psychological stress and burden are associated to constipation in children. To overcome functional constipation in children, a collaborative effort is required between parents, children, and the healthcare professional.

## Introduction

Functional constipation (FC) is one of the most prevalent gastrointestinal disorders in children, affecting up to 29.6% of the pediatric population worldwide [1]. According to the Rome IV criteria, functional constipation is defined when children exhibit at least two of the following conditions; less than three stools per week, a minimum of one incident of fecal incontinence, a history of severe stool retention or retentive posture, a background of difficult or painful bowel movements, a prior history of a large-diameter stool that could prevent the use of the toilet for at least two months [2]. At least one clinical sign of functional constipation is present in 20% of children and 0.3% with fecal incontinence [2,3]. Constipation in children has a complex pathophysiology which is influenced by interactions among several risk factors. The brain-gut axis appears to be a key factor in the pathogenesis of functional constipation because of its influence on pelvic floor and rectal function. Impaired coordination of the abdominal and pelvic floor muscles causes paradoxical contractions and inadequate anal relaxation during stool evacuation [2,3]. As a functional disorder, the development of constipation is influenced by a child´s psychosocial condition, even considered as one of important contributing factors [4]. Several studies have found a correlation between functional constipation and the psychological condition of children. School-aged children who experience functional constipation have aberrant oral habits which are considered as indirect indicators of psychological stress, such as lip sucking, finger sucking and nail biting [5]. A study on students in Sri Lanka who experienced stressful events at school or at home were more likely to experience functional constipation [5]. A study conducted by Ozokutan *et al*. demonstrates the relationship between functional constipation and child behavior, disturbed parent-child interactions, and stressful family situations [6]. Identifying a child´s psychosocial condition holistically could help with comprehending the family structure and parent-child connection [4]. Although, several studies about those role in functional constipation have been conducted, the studies were insufficient to draw firm conclusions. Therefore, in this systematic review, we intend to evaluate the relationship between psychological stress and constipation in children.

## Methods

**Review design:** our systematic review was conducted using the guidelines of Preferred Reporting Items for the Systematic Review and Meta-Analysis (PRISMA).

**Literature search:** we used three online databases as our study sources; PubMed, Cochrane library, and Google scholar. We limited the study search to studies published in the past five years, from 2018 to December 2022. The keyword we used for study searching was a combination of some keywords with Boolean operators consisting of “functional constipation”, “children”, “pediatric”, and “psychological stress”. Details of the search strategy in our study are presented in Appendix 1.

**Study eligibility:** we determined the study eligibility using inclusion criteria as follows: (1) a study that evaluates the relationship between psychological burden or psychological stress with functional constipation in children, (2) using a pediatric sample with an age range of 0-18 years old, (3) using sample with a functional constipation diagnosis based on a cluster of symptoms or the Rome criteria, (4) original article, and (5) published in full manuscript format in English.

**Figure 1:**
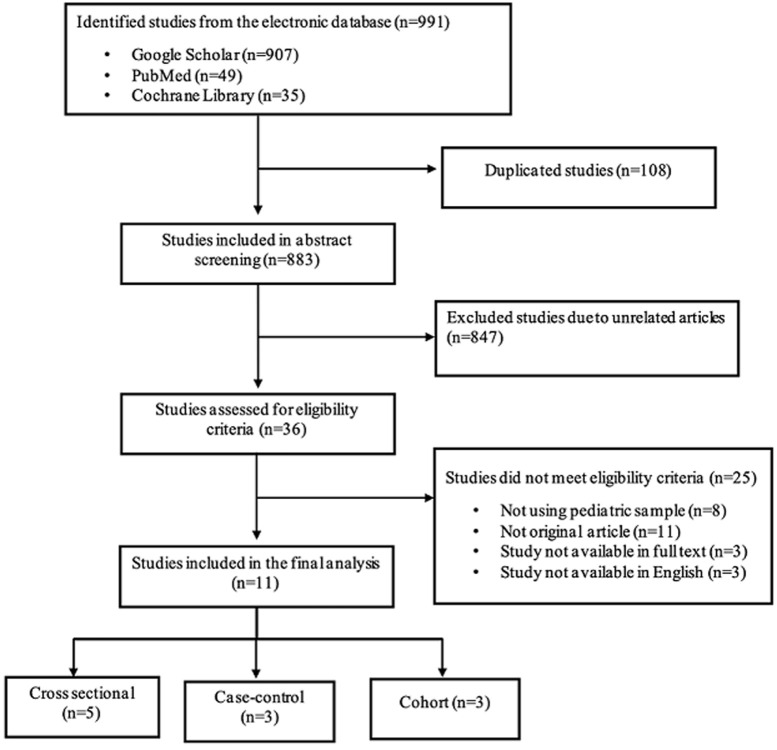
the PRISMA flowchart of our systematic review

**Screening and selection of articles:** two authors (FSG and BF) independently screened the abstracts from the collected articles. Disagreements of the abstracts reviewed were resolved by discussion a third authors (BH). The duplicate results were removed and unrelated articles were excluded from abstract screening. The selected studies were screened for the abstract to determine the applicability to our study subject. Studies that pass the screening step are then assessed for the full-text manuscript using eligibility criteria. Studies that met the eligibility criteria were then included in the data extraction and synthesis. All the study selections were carried out using The Preferred Reporting Items for the Systematic Review and Meta-Analysis (PRISMA) diagram, as seen in Figure 1.

**Study quality assessment:** a study quality assessment was done using The Joanna Briggs Institute (JBI) Critical Appraisal checklist for cross sectional, case-control, and cohort studies by two authors (IGNSP and IPGK). There are several assessments based on The JBI Critical Appraisal questions to assess the quality of the study. Assessment criteria are given a value of ‘yes’, ‘no’, ‘unclear’ or ‘not applicable’, and each criterion with a ‘yes’ score is assigned one point, and all other values score a zero; each study score is then calculated and summed up. Critical appraisal assesses studies that met the requirements and were carried out by researchers. If the research score is 50%, it meets the critical criteria appraisal; then studies were included in the inclusion criteria. Researchers excluded studies of low quality to avoid bias in the validity of the results and recommendations review [7,8].

**Synthesis of the study:** the selected articles that complied with the eligibility standards and quality assess were extracted by two authors (FSG and BH) using a Microsoft Excel. These data included authors, years of publication, origin country, study design, methods, sample size, range of age, gender distribution, diagnostic criteria for FC, intervention and control groups, assessed outcomes, and study findings from the studies included in the analysis. Extracted data were then used to complete the narrative synthesis. As qualitative research, this systematic review collects study information on the relationship between psychological stress and functional constipation in children.

## Current status of knowledge

**Study characteristics:** we discovered 991 studies that matched the search terms based on the findings of three electronic database search results. Eight hundred eighty-three studies were reviewed for abstracts to see if they were appropriate for the research topic after 108 duplicate studies were eliminated. Thirty-six studies were examined for eligibility after 847 were eliminated from the abstract screening due to unrelated articles. Only eleven studies from the final phase were included in the analysis because a total of 25 studies failed to meet the eligibility requirements, as indicated in Figure 1. Among the eleven studies, four cross-sectional studies, four case-control and three cohort studies were included. These studies were from several countries, such as Brazil, China, Germany, Indonesia, Iran, Japan, Netherlands, Sri Lanka, and the UK, were published up to December 2022, as stated in Table 1 [9-20].

**Table 1:** detailed study characteristics

Author	Study design	Sample size	Age range/ Mean age (years)	Male (%)	FC criteria	FC prevalence(%)	Identified psychological risk factor
dos Santos *et al*., 2021, Brazil [10]	Cross sectional	799	5-18	46.9	IV	20	Emotional symptoms (p<0.001) Conduct problems (p<0.001) Hyperactivity (p=0.08) Peer relationship problems (p=0.19)
Edan *et al*., 2022, Sri Lanka [11].	Cross sectional	165	0-17	69.7	IV	56.7	Types of psychological stressor found: fear of using the toilet, the start of school, new family members, verbal or physical abuse, death of family members, and family quarrels.
Equit *et al*., 2022, Germany. [12]	Case-control	40	5-12	70	III	62.5	Children with FC also exhibited a greater prevalence of psychiatric disorders in children, including ADHD, and oppositional defiant disorder, as well as considerably higher T-values for both externalizing and internalizing symptoms (p<0.001).
Joinson *et al*. 2018, UK.[13]	Cohort study	8,345	-	51.7	-	-	Difficult mood (OR=1.32), behavior problems (OR=1.48), temper tantrums (OR=1.89), lack of regular sleep routine (OR=2.09), and stressful life events (OR=1.32).
Niu *et al*., 2022, China. [14]	Case-control	432	42-6	52.31	IV	25	Parental conflict (OR=1.981), authoritarian family>2.481), anxiety control or temper control in children (OR=0.492). Conflict in relation between child and parents was significantly higher in FC group (P<0.001)
Oswari *et al*., 2018, Indonesia.[15]	Cross sectional	1,796	10-17	40.75	III	18.3	Constipation was significantly association with stressful life events like the father's alcoholism (OR 2.38), a close family member's severe illness (OR 2.49), divorce of parents (OR 2.18) the child being hospitalized for another illness (OR 2.09), being bullied at school (OR 2.43) and losing a parent's job (OR 1.74).
Sangari *et al*., 2022, Iran. [16]	Case-control	200	7.51 ± 1.91	59	-	-	Obsessive-compulsive disorder (OCD), generalized anxiety, and separation anxiety disorder all had considerably higher rates in the functional constipation group (P = 0.001), respectively.
Sawyer *et al*., 2022, UK. [17]	Cohort study	6,489	0-7	51.7	-	10.2	Antenatal maternal depression (p=0.004, OR=1.46), antenatal maternal anxiety (p<0.001, OR=1.57), post-natal maternal anxiety (p<0.001, OR=1.63), post-natal maternal depression (p=0.017, OR=1.33).
Walter *et al*., 2019, Netherlands. [18]	Cross sectional	1,113	6.5-48 months	49.7	III	8	Physical or verbal violence (p=0,046), children whose mother suffered physical/verbal violence (0=0.051), loans of family (p=0.770), and parents relationship (p=0.751)
Yamada *et al*., 2021, Japan [19].	Cohort study	5,540	9-10	51.3	-	4.7	Adolescents irritability (OR 1.59) and infrequent child-parents interaction (OR 0.91)
Zafari *et al*., 2021, Iran [20].	Case Control	247	4-12	50	III	-	ADHD was significantly correlated to functional constipation in children (p=0.0001)

Abbreviation: ADHD= Attention Deficit Hyperactive Disorder; FC= Functional Constipation; OR= Odds Ratio; UK= United Kingdom

**Study quality:** quality assessments of the study were done by two authors (FSG and BF) to avoid bias. The assessment was carried out using a checklist from Joanna Briggs Institute according to the study design. Criteria for cross sectional study consist of eight parameters, while for case-control and cohort study consists of ten and eleven parameters [7-10]. The detailed assessment of study quality is shown in (Table 2, Table 2.1, Table 2.2). All studies are considered good quality based on the study quality evaluation.

**Table 2:** quality assessment of cross-sectional study

Criteria	dos Santos *et al*., 2021[10]	Edan *et al*., 2022[11]	Oswari *et al*., 2018[15]	Walter *et al*., 2019[13]
1) Were the criteria for inclusion in the sample clearly defined?	1	1	1	1
2) Were the study subjects and the setting described in detail?	1	1	1	1
3)Was the exposure measured validly and reliably?	1	1	1	1
4) Were objective, standard criteria used to measure the condition?	1	1	1	1
5) Was exposure measured the same way for cases and controls?	0	0	0	0
6) Were strategies to deal with confounding factors stated?	0	0	0	0
7) Were the outcomes measured validly and reliably?	1	1	1	1
8) Was appropriate statistical analysis used?	1	1	1	1
Total	6	6	6	6

Note: Yes=1 point; No, unclear, or unapplicable=0 points.

**Table 2.1:** quality assessment of case study

Criteria	Equit *et al*., 2022[12]	Sangari *et al*., 2022[16]	Zafari *et al*., 2021[20]
1)Were the groups comparable other than the presence of disease in cases or the absence of disease in controls?	1	1	1
2) Were cases and controls matched appropriately?	1	1	1
3)Were the same criteria used to identify cases and controls?	1	1	1
4)Was exposure measured in a standard, valid and reliable way?	1	1	1
5)Was exposure measured the same way for cases and controls?	1	1	1
6)Were confounding factors identified?	0	0	0
7)Were strategies to deal with confounding factors stated?	0	0	0
8)Were outcomes assessed in a standard, valid and reliable way for cases and controls?	1	1	1
9)Was the exposure period of interest long enough to be meaningful?	1	1	1
10)Was appropriate statistical analysis used?	1	1	1
**Total**	8	8	8

Note: Yes=1 point; No, unclear, or unapplicable=0 points.

**Table 2.2:** quality assessment of cohort study

Criteria	Joinson *et al*., 2018 [13]	Sawyer *et al*., 2022 [17]	Yamada *et al*., 2021[19]
1)Were the two groups similar and recruited from the same population?	1	1	1
2)Were the exposures measured similarly to assign people to both exposed and unexposed groups?	1	1	1
3)Was the exposure measured validly and reliably?	1	1	1
4) Were confounding factors identified?	1	1	0
5)Were strategies to deal with confounding factors stated?	1	1	0
6)Were the groups/participants free of the outcome at the start of the study (or at the moment of exposure)?	1	1	1
7)Were the outcomes measured validly and reliably?	1	1	1
8)Was the follow-up time reported sufficiently long enough for outcomes to occur?	1	1	1
9) Was follow-up complete, and if not, were the reasons to lose to follow-up described and explored?	1	1	1
10)Were strategies to address incomplete follow-up utilized?	1	1	1
11)Was appropriate statistical analysis used?	1	1	1
**Total**	11	11	9

Note: Yes=1 point; No, unclear, or unapplicable=0 points.

**Patient characteristics:** all eleven studies included 25,116 pediatric patients as a study sample, with an age range from 2 months to 17 years old [9-20]. Seven studies (63.6%) are mostly made up of males [10-13,15,16,18]. Rome criteria were utilized in most research (63.6%); three studies used Rome IV [9,10,13] and four used Rome III criteria to diagnose FC [11,14,17,19]. The remainder defined FC using based on a cluster of symptoms [12,15,16,18]. We found varied FC prevalence, ranging from 4.7-62.5%. The highest FC prevalence was found in a case-control study by Equity *et al*. [9] and the lowest was found in a cohort study in Japan by Yamada *et al*.as seen in Table 1 [18].

**Identified psychological risk factors for constipation in children:** we have analyzed several risk factors related to pediatric constipation based on the included studies. We can classify them into family-related stressors, school-related stressors, exposure to stressful events, stress related to psychological disorders, and other factors (Table 1).

**Family-related stressor :** a cross sectional study by Edan *et al*. stated that psychological stress are significantly associated to pediatric constipation, in which family-related stressors such as a new family member, death of family members, and family quarrels are included [10]. The other study by Joinson *et al*. stated that irregular sleep routine significantly related to FC with OR 2.09 and [12]. A case-control study by Niu *et al*. found that stressor for children, such as parental conflict, and authoritarian family style has significant relationships with constipation in children with OR 1.981, and 2.481, respectively. They also showed that conflict of child-parents relationship was significantly higher in FC group (P<0.001) [14]. Study in Indonesia by Oswari *et al*. also found that children whose parents lost jobs were also significantly associated to pediatric constipation with OR 1.74 [15]. While a study by Walter *et al*. found that stressful life event in family including physical or verbal violence (p=0.046), loans of family (p=0.770), and parents relationship (p=0.751) do not statistically significant as predispose children to develop FC [18]. Similar result showed by Yamada *et al*. in the Japanese pediatric population that frequency of child-parents interaction were not associate with constipation (p=0.874) [18].

**School related stressor:** a study by Edan *et al*. found that fear of using the toilet and the start of school were included in a psychological stressor that caused FC in children with p <0.001 [10]. While a study by Oswari *et al*. found that being bullied at school also has a significant associated with FC with OR 2.43 [14]. A cross sectional study by Dos Santos *et al*. showed that no correlation was found between peer relationship problems and constipation in children (p=0.19) [9].

**Exposure to stressful life events:** exposure to stressful life events is found to be related to pediatric constipation in our included study. A study by Joinson *et al*. found that children with stressful life events are 1.23 times more likely to develop FC than children without stressful life events [12]. A cross sectional study conducted in Indonesia by Oswari *et al*. found that stressful life events such as paternal alcoholism, a close family member's severe illness, and the child being hospitalized for another illness significantly associated to constipation in children with OR 2.38, 2.49, and 2.09, respectively [14].

**Stress related to psychological disorders:** stress related to psychological disorders are also strongly associated with the occurrence of constipation in children. First is a study by dos Santos *et al*.concluded that children with FC had more emotional and behavioral problems than non-constipated children. They found that children with emotional symptoms, conduct problems, and hyperactivity were statistically significantly associated to pediatric constipation [9]. Additionally, Equit *et al*. found that children who experience fecal incontinence and constipation show more psychological symptoms and psychiatric disorders such as ADHD and oppositional defiant disorder [11]. A prospective study by Yamada *et al*. found that adolescents who experience irritate continously were more developed constipation within three years (OR 1.59) [18]. A study by Joinson *et al*.found that increased risks of constipation and soiling were linked to difficult temperament (OR=1.89), and emotional or behavioral issues (OR=1.32; OR=1.48) [12]. A case control study by Zafari *et al*. found that children with FC have a higher risk of ADHD (OR 3.082) [19]. While a study by Niu *et al*. showed that anxiety control or temper control in children with constipation were statistically lower than children without constipation (p<0.001) [13]. The last study by Sangari *et al*. that was conducted in Iran found that functional constipation in children statistically associated with some type of anxiety disorder [15].

**Other factors:** other factors that were also found to be related to constipation in children were antenatal and post-natal maternal anxiety, as stated in a study by Sawyer *et al*. conducted in the UK. They found that the likelihood of incontinence or constipation in children exposed to maternal post-natal psychopathology was higher, and maternal anxiety was more strongly associated with these outcomes than maternal depression [16].

Our systematic review gathered information on the relationship between psychological stress and functional constipation as one of the most common Functional Gastrointestinal Disorders (FGID) found in children [2,3]. We classify psychological stressors in children into five broad spectrums, family-related stressors, school-related stressors, exposure to stressor life events, stress related to psychological disorders, and other factors. Family-related stressors are one of the most researched psychological variables. Five of eleven studies found that FC in children was associated with family-related stressors [10,12-14,18], one study found no associations [17], and the remainders did not evaluate family-related stressor but other psychological variables. Poor child-parent relationship is one of the main stressors in the family. Children who scored poorly on autonomy through the parenting style questionnaire had fewer bowel movements every day [13]. Authoritarian parents tend to over-regulate their children's autonomy while favoring their obedience [13].

School-related problems also associated with functional constipation in children [9,13]. The stressor can be bullying at school [13] and aversion to using toilets [9]. Bullying is a stressful event that activate neurohormonal stress response through hypothalamic-pituitary-adrenal (HPA) axis which delaying the colonic transit [21,22]. Aversion of using the restroom causes stool withholding, impaction fecal in the rectum, intestinal dilatation, decreased sensation, and slower peristaltism [11]. In contrast, peer-relationship problem was not associated with functional constipation in children [10]. Emotional symptoms such as difficult mood, temper tantrums, and anxiety are psychological disorders that have been reported to be significantly related to functional constipation in children [8,11,12,15]. Besides effect on their physical health, long-term exposure to stress in children has consequences for a significant psychological load [22]. Stress itself is a physical or psychological condition resulting from an immediate threat to a child´s homeostasis, either posed by external or internal events [22]. Stress triggers adaptive reactions, but can become “internalized” when unaddressed, which can cause anxiety and other psychological issues. Symptoms are expressed via pathways involving the central and enteric nervous systems. Anxiety cause distortion of cognitive/perceptual, smooth muscles, and striated muscles function. In fact, extreme anxiety can affect the function of smooth muscle and striated muscle simultaneously, as found in constipation and Irritable Bowel Syndrome (IBS) [14,20]. Individuals with functional constipation lacked efficient coping skills, which led to internalization of stress and restriction of colonic motility.

A link was identified between psychological anxiety and pelvic floor tension. Blood flow from the rectal mucosa to the intestine and the extrinsic nerve increases [2,23]. Functional magnetic resonance imaging (MRI) studies have demonstrated a linked between functional gastrointestinal disorders with activation of the anterior midcingulate and posterior cingulate, as well as deactivation of the anterior cingulate cortical supragenual region, which linked to downregulation of pain signals in children who have been exposed to abuse [24]. Abuse and other pathological stressors can alter the amygdala and cingulate cortex, thus affecting the function of the central nervous system and peripheral nervous system receptors remain for a long time [25]. Changes in gut microbiome composition, autonomic nervous system activity, neuroendocrine responses, regional brain activity, brain-gut axis, and hypothalamic-pituitary-adrenal axis may lead to lower bowel dysfunction and functional constipation [24,25]. Stress is a global psychological problem, including in children. Various factors as contributors to stress are found in children who experience constipation. However, it is still unclear whether constipation causes children to experience stress and exacerbate constipation or the stress experienced by children causes constipation. Although this systematic review showed a link between psychological stress and functional constipation in children, it is still difficult to determine definitively a causal relationship between the two, since several studies used a retrospective design. Few studies are not using Rome criteria as an established criteria to diagnose functional constipation in children. Even so, psychosocial aspects still need attention by doctors who take care of children with constipation [20,21]. Therefore, it is recommended to conduct more longitudinal studies in the future and continue with a meta-analysis study so that the causality of variables can be analyzed more comprehensively.

## Conclusion

Based on this systematic review, we can conclude that stress and psychological burden are associated with constipation in children. Family-related stressors, school-related stressors, exposure to stressful life events, stress-related psychological disorders, and other variables can cause psychological stress in children. Taking care children with functional constipation must pay attention to the child's psychological factors that may appear or have even appeared. Effective communication is needed between parents, children, healthcare professional, and the surrounding community in taking care children with functional constipation.

### What is known about this topic

- *Most constipation in children is a functional disorder that has a complex pathophysiology which is influenced by interactions among several risk factors;*
- *The development of constipation is influenced by a child´s psychosocial condition*.

### What this study adds

- *Association between psychological stress and constipation in children;*
- *We classify psychological stressors in children into five broad spectrums, family-related stressors, school-related stressors, exposure to stressful life events, stress-related psychological disorders, and other factors*.
